# Dual-Mode Quantitative Immunochromatographic Assay for Highly Sensitive On-Site Detection of Ciprofloxacin in Fish Products

**DOI:** 10.3390/foods14071132

**Published:** 2025-03-25

**Authors:** Junqi Shen, Zhengyi Cai, Cheng Zhang, Xinyue Feng, Chenzhi Zhang, Huan Zhao, Chuanlin Yin, Bo Wang, Xiaoping Yu, Biao Zhang

**Affiliations:** 1 Key Laboratory of Microbiological Metrology, Measurement & Bio-Product Quality Security, State Administration for Market Regulation, College of Life Sciences, China Jiliang University, Hangzhou 310018, China; shenjunqi@cjlu.edu.cn (J.S.); s24090710003@cjlu.edu.cn (Z.C.); 19548195033@163.com (C.Z.); fxy18767053369@163.com (X.F.); luocheng2026@163.com (C.Z.); kroyihuan@163.com (H.Z.); yxp@cjlu.edu.cn (X.Y.); 2 College of Life Sciences, China Jiliang University, Hangzhou 310018, China; 3 College of Food Science and Engineering, Yangzhou University, Yangzhou 225009, China; wb@yzu.edu.cn

**Keywords:** dual-mode quantitative immunochromatographic assay, photothermal immunoassay, portable reader, ciprofloxacin, PVP@Pd, aquatic product safety

## Abstract

Ciprofloxacin has been extensively utilized in aquaculture due to its remarkable efficacy in preventing and treating bacterial infections in fish animals. However, the widespread application of ciprofloxacin has led to significant residue accumulation, necessitating the development of rapid, sensitive and specific detection methods. In this study, we developed a novel dual-mode quantitative immunochromatographic assay based on a portable reader and a photothermal instrument, enabling on-site ciprofloxacin detection. Under optimized conditions, the portable reader mode (Mode 1) achieved a detection range of 0.1–100.0 ng/L with a limit of detection (LOD) of 0.1 ng/mL. The photothermal instrument mode (Mode 2) achieved a detection range of 0.1–500.0 ng/mL with an LOD of 0.1 ng/mL. The sensitivity and accuracy of the method were validated using an Enzyme-Linked Immunosorbent Assay. This developed method successfully detected ciprofloxacin residues in samples of *Parabramis pekinensis*, *Larimichthys crocea*, *Channa argus*, *Carassius auratus* and *Micropterus salmoides*, with satisfactory recovery rates. The results demonstrated excellent specificity and applicability across various fish product matrices, offering a reliable and efficient solution for the on-site monitoring of ciprofloxacin residues in fish products.

## 1. Introduction

Ciprofloxacin (CIP) is a third-generation synthetic fluoroquinolone antimicrobial agent [1], active against both Gram-negative and Gram-positive pathogens [2]. It exerts its sterilizing effect by targeting bacterial topoisomerases II (gyrase) and IV to inhibit their control of intracellular supercoiling, leading to cell death [3,4]. CIP is clinically recommended for a variety of bacterial infections and can also be used to treat infections in animals. Quinolones were introduced in the late 1980s to treat food animals. The use of quinolones now extends to domestic animals such as chickens, cattle and pigs, as well as croaker, shrimp, bullfrogs and other aquatic species [5,6]. Due to its high efficiency and other advantages, CIP is used to treat poultry colibacillosis in China [7]. According to a WHO report, quinolones accounted for 2.7% of the veterinary antibiotics used between 2015 and 2017 [8,9]. According to thane FDA report, sales of quinolone in the United States increased from 15.1 tons in 2013 to 24.5 tons in 2019 [10]. However, the application of high doses leads to significant residues in various environmental and animal-derived products, posing potential risks to human health, including the proliferation of drug-resistant bacteria, the disruption of intestinal microbiota, DNA damage and potential carcinogenic and teratogenic effects [11].

More than 75% of CIP is excreted unmetabolized into the environment, contaminating surface water, groundwater and drinking water. These substances enter wastewater treatment plants, which are unable to remove them [1]. In addition to water samples, CIP is frequently detected in food products such as fish and domestic animals. It has been reported that 17 kinds of antibiotics have been identified in large yellow croaker in five breeding sites along the coast of Zhejiang, China. The average residual amount of enrofloxacin and CIP in their edible parts was 23.18 μg/kg [12]. It has been reported that the detection rate of CIP in samples of large black fish, freshwater shrimp, chicken, eggs and pork is 11.6% [13]. Due to the drug resistance and various potential health hazards that CIP may cause in humans [14,15,16], quinolones are not allowed in aquaculture in the United States [17]. However, in countries such as the United Kingdom, Poland, China and Brazil, they are authorized for use in poultry and other food animals [18,19]. In China, there is no maximum residue limit (MRL) for CIP in food according to GB 31650-2019 (Chinese National standard) [20]. Therefore, the development of an animal-derived-product residue detection method for CIP is of great significance to protecting human health and the environment.

At present, many methods for the detection of CIP have been reported, including thin-layer chromatography (TLC) [21], high-performance liquid chromatography (HPLC) [22,23], liquid chromatography–mass spectrometry (LC-MS) [24], Enzyme-Linked Immunosorbent Assays (ELISAs) [25] and UV–Visible spectrometers [26]. These methods have good sensitivities and limits of detection (LODs) for CIP in samples. However, due to complex matrix interference, these detection methods require a cumbersome and time-consuming purification and enrichment pretreatment process. At the same time, the above methods are based on large instruments, require professional operators, have high maintenance costs and are difficult to promote and apply in environments with limited resources [27,28]. SERS [29,30], electrochemistry [31,32] and other new detection technologies have also been widely studied, but the shortcomings of their high costs, low applications and high complexity of instruments are difficult to overcome [33].

Therefore, it is necessary to establish a high sensitivity, simple and rapid CIP detection method based on low-cost portable equipment. Immunochromatography is becoming one of the most promising trace detection applications in resource-limited environments due to its high sensitivity, real-time analysis and product commercialization. Paper-based immunoassays have the characteristics of low cost, convenient operation and less sample consumption, so they have been widely studied and applied [34,35]. In order to improve their sensitivity and give full play to their advantages with limited resources, many researchers have constructed a high-sensitivity multi-mode output immunoassay by synthesizing new nanosignal materials, optimizing detection methods and combining the output functions of the device [36,37,38].

In this study, anti-CIP monoclonal antibodies (CIP antibodies) were conjugated onto the surfaces of PVP@Pd NPs to serve as a signal probe (PVP@Pd NPs-mAb), enabling dual-signal output in both a portable reader and a photothermal instrument. The applicability of the established immunochromatographic method was evaluated by measuring the concentration of CIP in real samples, ensuring its robustness and reliability across different analytical conditions. The detected results indicate that the proposed method exhibits excellent specificity and high applicability across various food matrices, demonstrating its potential for practical applications in food safety monitoring. A new dual-mode strategy is provided and used for the sensitive and rapid detection of CIP in real samples in this work, offering an efficient and reliable approach for on-site screening and regulatory surveillance.

## 2. Materials and Methods

### 2.1. Materials and Instruments

The drug standards and Tween-20 were purchased from Maclin Biochemical Technology (Shanghai, China). PVP 40, K_3_[Fe(CN)_6_], Na_2_[PdCl_4_] and bovine serum albumin (BSA) were purchased from Sigma Aldrich (Shanghai, China). Goat anti-mouse secondary antibodies IgG (anti-antibodies) were purchased from Jackson Immuno Research Laboratories (West Grove, PA, USA). The anti-CIP monoclonal antibodies (CIP antibodies) and CIP antigens were purchased from Kejie Industrial Development (Shenzhen, China). Nitrocellulose membranes (NC membranes), conjugate pads, absorbent pads and PVC backing cards were purchased from Jinbiao Biotechnology (Shanghai, China). Real fish samples were purchased from local farmers’ markets (Hangzhou, China).

A DNP-9052B electric thermostatic incubator was purchased from Mingde Instrument (Hangzhou, China). A HM3035 XYZ three-dimensional film gold sprayer was purchased from Jinbiao Biotechnology (Shanghai, China). A BIO-VINOSTECH programmable single-knife cutting machine was purchased from Hangan Electronic Technology (Shanghai, China).

### 2.2. Preparation of PVP@Pd NPs and PVP@Pd NPs-mAb

PVP@Pd NPs were prepared using the method referred to the literature with some modifications [39]. Briefly, 6 g of PVP40, 226.8 mg of K_3_[Fe(CN)_6_] and 80 mL of 0.01 M HCl solution were, respectively, added into a round-bottomed flask. The mixed solution was stirred to dissolve them. Then, the mixed solution was heated to 80 °C and reacted for 20 h. After centrifugation (12,000 rpm, 15 min), PVP NPs were obtained and washed with deionized water. The pure PVP NPs were reconstituted to 1.0 mL. A total of 20 μL of 0.5 mM Na_2_[PdCl_4_] and 1 mL of 0.1 mM NaBH_4_ were added into an appropriate amount of PVP NPs. Subsequently, the mixed solution was stirred continuously at room temperature for 1 h. PVP@Pd NPs were obtained by centrifugation (12,000 rpm, 15 min) for later use.

PVP@Pd NPs-mAb were prepared using the method referred to in the literature [40]. The optimization method was to add 1 µL of CIP antibodies (7.3 mg/mL) and incubate at 4 °C for 1 h. Then, 20 µL of bovine serum albumin (BSA) aqueous solution (20% *w*/*v*) was added and incubated at 25 °C for 30 min. After being centrifuged twice (12,000 rpm, 15 min), the PVP@Pd NPs-mAb conjugate was obtained by resuspending the precipitate in a buffer solution.

### 2.3. Procedure of Dual-Mode Quantitative Immunochromatographic Assay

The immunochromatographic strips were fabricated following our established protocol, with some modifications: A sample pad, NC membrane and absorbent pad were sequentially laminated onto a PVC backing. The appropriate concentrations of CIP antigens and anti-antibodies were then plotted on the T and C lines, respectively. The whole piece was dried overnight in a constant temperature drying oven. A test strip with a width of 0.40 mm was prepared by using the test paper mechanism. We assembled the test strip in the card slot and stores it at room temperature until use.

The construction process of the dual-mode detection platform based on immunochromatographic strips was as follows: 100.0 μL CIP standard solution/sample extract solution (concentration gradient: 0, 0.1, 0.5, 1.0, 10.0, 50.0, 100.0, 500.0 ng/mL) was mixed with a 10.0 μL solution containing 10.0% Tween-20 and 20.0 μL signal probe (PVP@Pd NPs). After transferring the mixture to the strip sample pad, the result could be visually interpreted within 5 min. After the chromatogram was completely terminated, semi-quantitative analysis was carried out through the color rendering intensity ratio of the T line and C line. When the color rendering of the T line was weaker than that of the C line and did not disappear completely, the corresponding minimum concentration was defined as the visual detection limit. Then, the test strip was placed into a portable reader, where the T line color intensity was detected and converted into a grayscale value. This grayscale value reflected the aggregation of nanoparticles at the T line, which was directly related to the concentration of the target analyte. A linear standard curve (Mode 1) was established to correlate the grayscale values with the analyte concentrations, enabling quantitative detection. The photothermal instrument platform was mutually verified with Mode 1 by irradiating the test strip with an 808 nm laser and obtaining the temperature through a thermal imager. Then, the temperature was linearly fitted to establish a standard curve for the other CIP detection method (Mode 2), and the photothermal instrument detection method for CIP was established.

### 2.4. Application in Real Sample

The pretreatment of the samples (*Parabramis pekinensis*, *Larimichthys crocea*, *Channa argus*, *Carassius auratus* and *Micropterus salmoides*) was conducted in strict accordance with Chinese local standard DB34/T 3637-2020 [41]. Briefly, 2 g of homogenized tissue was extracted with 3 mL ethyl acetate via 3 min of vigorous vortexing, followed by centrifugation at 4000 rpm for 5 min. A 2 mL aliquot of the supernatant was collected and evaporated to near-dryness under a nitrogen stream at 50–60 °C. The residue was reconstituted with 0.3 mL n-hexane and 0.3 mL phosphate-buffered saline (PBS, 0.01 M, pH 7.4) through thorough mixing. For spiked recovery tests, CIP standard solutions were introduced to create concentration gradients (0.1–500 ng/mL). HPLC confirmation verified the absence of endogenous CIP residues in all blank samples prior to fortification.

### 2.5. Instrument Validation

To validate the accuracy of the developed method in the real samples, CIP standard solutions were spiked into negative samples to prepare labeled samples with concentration gradients (0, 10, 20 ng/mL). Detection was performed on *Parabramis pekinensis*, *Larimichthys crocea*, *Channa argus*, *Carassius auratus* and *Micropterus salmoides* samples using an ELISA. The pretreatment procedures and experimental conditions for the ELISA were modified based on the DB34/T 821-2024 (Chinese local standard) [42]. Dual-mode quantitative immunochromatographic analysis and the ELISA were conducted simultaneously, with triplicate measurements for each spiked concentration. The optimal operating conditions and S-shaped curve of ciprofloxacin in PBS from the ELISA are shown in Table S1 and Figure S1.

## 3. Results

### 3.1. Principle of Dual-Mode Quantitative Immunochromatographic Assay

**Figure 1:**
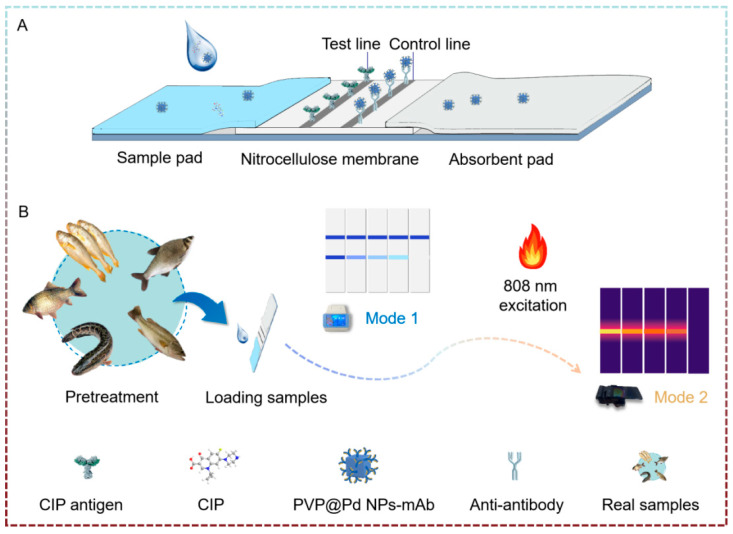
Schematic diagram of dual-mode quantitative immunochromatographic assay for detection of CIP. Competitive immunochromatographic assay (**A**) and detection output mode based on portable reader and photothermal instrument (**B**).

The schematic diagram is shown in Figure 1A. After mixing PVP@Pd NPs-mAb with the sample solution, the mixture is applied to the sample pad, and chromatography is carried out on the NC membrane. The presence of CIP in the sample affects the interaction between PVP@Pd NPs-mAb, the CIP antigen and the antibody on the test strip. In the absence of CIP, PVP@Pd NPs-mAb specifically binds to the CIP antigen on the T line, forming a visible blue band. Meanwhile, PVP@Pd NPs-mAb also binds to the secondary antibody on the C line, producing a blue band, indicating a negative result. For samples containing CIP, the CIP molecules bind to PVP@Pd NPs-mAb, reducing the availability of unbound PVP@Pd NPs-mAb to interact with the CIP antigen on the T line. Consequently, the intensity of the band on the T line decreases, indicating a positive result. Regardless of the CIP concentration, the C line always shows a color band as a quality control indicator. If no color is present on the C line, the test strip is invalid.

In portable reader mode, the test strip is inserted into the reader, where the T line color intensity is measured and converted into a grayscale value by an algorithm. This value, which reflects the amount of gold nanoparticle aggregation at the T line, is then correlated with the CIP concentration through a linear standard curve (Mode 1), providing the basis for quantitative analysis. The photothermal instrument platform can be mutually verified with Mode 1 by irradiating the test strip with an 808 nm laser and obtaining the temperature through a thermal imager. Then, the temperature is linearly fitted to establish a standard curve for the other CIP detection method (Mode 2), and the photothermal instrument detection method for CIP is established (Figure 1B).

### 3.2. Characterization of PVP@Pd NPs and PVP@Pd NPs-mAb

**Figure 2:**
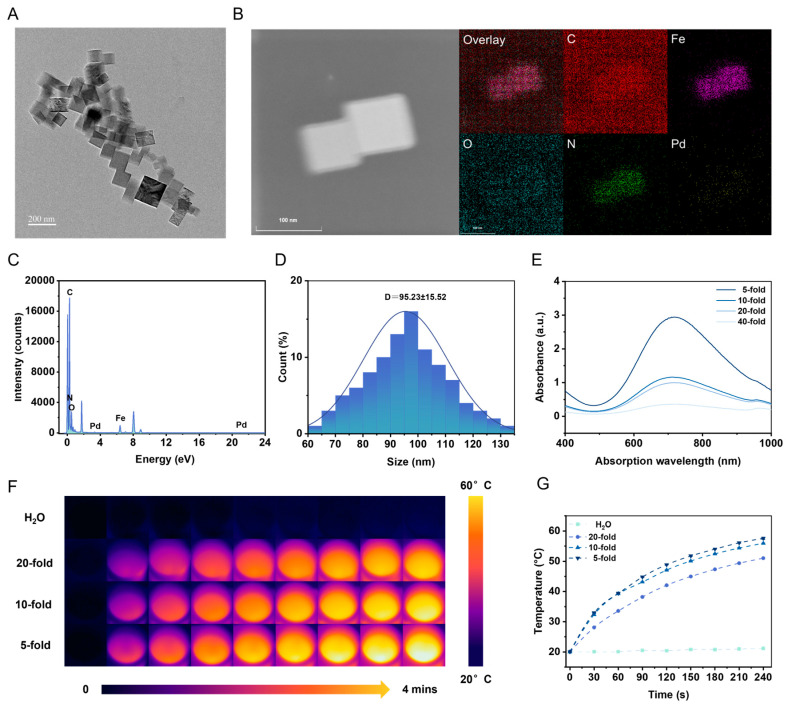
The characterization of the PVP@Pd NPs. The TEM image (**A**), element mapping (**B**), EDX spectra (**C**), size distribution (**D**) and UV-vis-NIR absorbance spectra at different dilutions (**E**) of PVP@Pd NPs. Photothermal images (**F**) and the corresponding photothermal temperatures (**G**) under laser irradiation from 0 to 5 min in the presence of PVP@Pd NPs from 5-fold dilution to 20-fold dilution.

The synthesized PVP@Pd NPs exhibited a homogeneous deep-blue coloration with a clear solution free of suspensions or precipitates. Transmission Electron Microscopy (TEM) analysis revealed uniformly distributed PVP@Pd NPs with excellent dispersibility (Figure 2A). High-Angle Annular Dark-Field Scanning Transmission Electron Microscopy (HAADF-STEM) imaging confirmed the homogeneous distribution of Pd and Pt within the nanoparticles (Figure 2B), while Energy Dispersive Spectroscopy (EDS) spectra displayed characteristic peaks of Pd and Pt (Figure 2C). The PVP@Pd NPs demonstrated an average diameter of 95.23 nm, with a superior dispersion stability (Figure 2D). Ultraviolet–Visible–Near-Infrared (UV-vis-NIR) absorption spectroscopy indicated that the absorbance increased as the reagent concentration increased (Figure 2E).

Under 808 nm laser irradiation, the PVP@Pd NPs exhibited remarkable photothermal conversion efficiency. Systematic investigation of the PVP@Pd NPs at different dilution factors revealed concentration-dependent temperature elevation during irradiation (0–5 min), whereas the control group (pure water) showed negligible temperature variation (Figure 2F). Quantitative analysis demonstrated that 5-fold diluted PVP@Pd NPs could elevate the temperature from 20.0 °C to 57.6 °C after 5 min of NIR irradiation, in contrast to pure water, which only increased the temperature from 20.0 °C to 21.1 °C under identical conditions (Figure 2G).

### 3.3. Optimization of Experimental Conditions

**Figure 3:**
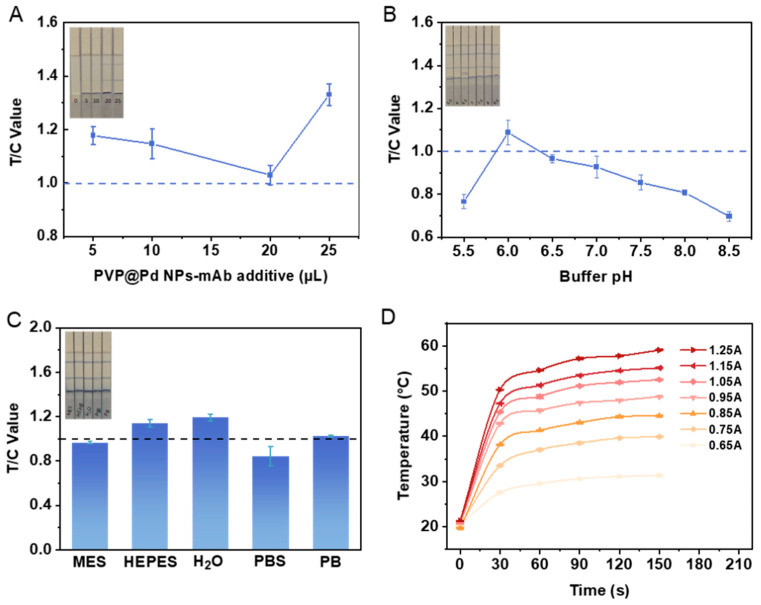
Optimization results for experimental conditions. Optimization of PVP@Pd NPs-mAb additive amount (**A**), buffer pH value (**B**), buffer type (**C**) and laser irradiation current (**D**). (**A**–**C**) each contain images of test strips in upper left corner.

To achieve high-sensitivity detection in immunochromatographic strips, this study systematically optimized four critical parameters: signal probe loading volume, buffer pH, buffer type and laser irradiation current and time. Initially, by comparing T/C values at different PVP@Pd NPs-mAb loading volumes (5.0, 10.0, 20.0, 25.0 µL), the optimal loading volume was determined to be 20.0 µL (Figure 3A). Subsequently, the buffer pH was adjusted using 0.2 M K_2_CO_3_ to various values (5.5, 6.0, 6.5, 7.0, 7.5, 8.0, 8.5), with pH 6.0 demonstrating the optimal T/C value (Figure 3B). Following this, different buffer systems (MES, HEPES, H_2_O, PBS, PB) were compared at pH 6.0, revealing that the PB buffer yielded T/C values closest to 1 (Figure 3C). Finally, we evaluated temperature variations under different laser irradiation currents (0.65, 0.75, 0.85, 0.95, 1.05, 1.15, 1.25 A) and different times (30 s, 60 s, 90 s, 120 s, 150 s). Currents from 0.95 A to 1.25 A caused rapid temperature increases followed by plateaus, while currents from 0.65 A to 0.75 A showed insufficient heating. After 120 s of irradiation, the temperature entered a plateau. Considering both safety and reproducibility, 0.85 A was selected as the optimal irradiation current and laser irradiation time (Figure 3D).

### 3.4. Performance of Dual-Mode Quantitative Immunochromatographic Assay

**Figure 4:**
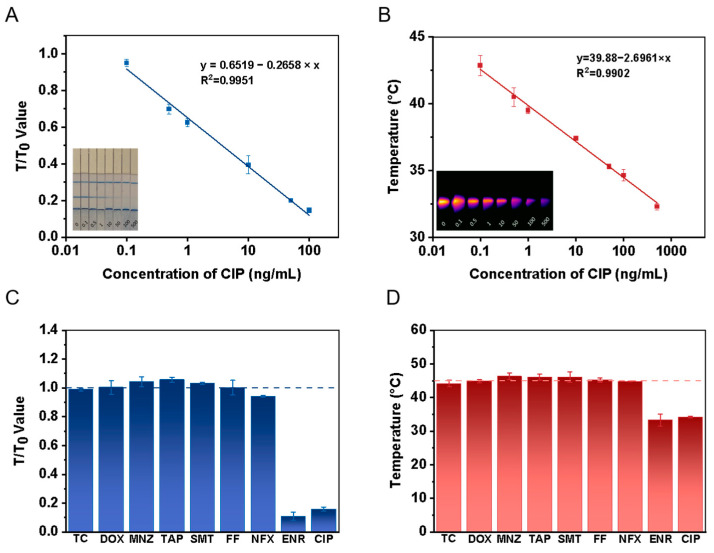
Detection output mode based on portable reader and photothermal instrument. Standard curve in portable reader mode (**A**). Standard curve in photothermal instrument mode (**B**). Specificity results for CIP and other antibiotics in portable reader mode (**C**). Specificity results for CIP and other antibiotics in photothermal instrument mode (**D**). (**A**,**B**) each contain images of test strips in lower right corners.

After optimizing a series of working parameters, the dual-mode quantitative immunochromatographic assay was performed. The prepared CIP test strips were used to detect diluted solutions of standard substances with concentrations of 0, 0.1, 0.5, 1.0, 10.0, 50.0, 100.0 and 500.0 ng/mL, and the sensitivity and detection limits of the test strips in the different output modes were determined. As shown in Figure 4A, a standard curve for CIP was established using a portable reader, with a linear range of 0.1–100.0 ng/mL. The linear equation was y = 0.6519 − 0.2658 × x, and the correlation coefficient (R^2^) was 0.9951. The LOD of CIP was 0.1 ng/mL. A CIP standard curve was also established by 808 nm laser irradiation for 120 s, based on the increase in temperature. The linear equation was y = 39.8759 ~ 2.6961 × x, with a correlation coefficient (R^2^) of 0.9902, and the LOD of CIP was 0.1 ng/mL (Figure 4B). These results confirm that the dual-mode quantitative immunochromatographic assay test strips prepared in this study exhibit high sensitivity and can be used for the quantitative detection of CIP. In comparison to previous methods, such as using HPLC, immunosensors, electrochemical sensors, and fluorescences, this new method offers several advantages, including a lower detection limit, higher sensitivity, broader detection range, enhanced dual-signal output capability and the flexibility to select the signal output mode as needed (Table S2).

It is essential to evaluate the specificity of the dual-mode quantitative immunochromatographic assay for its application. The prepared test strips were used to detect antibiotics with functions similar to CIP or commonly used antibiotics, including tetracycline (TC), doxycycline (DOX), metronidazole (MNZ), tilmicosin (TAP), sulfamethoxazole (SMT), florfenicol (FF), norfloxacin (NFX) and enrofloxacin (ENR). Then, 100.0 ng/mL of the above-mentioned standard substances were added to the sample pads of strips to evaluate the application of the dual-mode quantitative immunochromatographic assays. In the portable reader mode, the ratios (T/T_0_) of the T lines of the test strips with the above-mentioned standard substances and without added CIP were measured and compared (Figure 4C). In the photothermal instrument mode, the temperature of the T line under 808 nm laser irradiation for 120 s was measured and compared to the temperature of the T line of the test strip without added CIP (Figure 4D). The results show that CIP exhibits good specificity compared to TC, DOX, MNZ, TAP, SMT, FF and NFX. Given that CIP is the major metabolite of ENR [43,44], and both share similar structural characteristics, this structural similarity could lead to cross-reactivity at the T line of the immunochromatographic test strip. Based on this feature, the developed assay is applicable not only for CIP detection but also for ENR detection. Experimental results demonstrate that the established immunoassay method exhibits good specificity for CIP.

### 3.5. Practical Application Results for Real Samples

The dual-mode quantitative immunochromatographic assay was evaluated for detecting CIP residues in fish product samples, including *Parabramis pekinensis*, *Larimichthys crocea*, *Channa argus*, *Carassius auratus* and *Micropterus salmoides* at target concentrations of 10.0 and 20.0 µg/L. As shown in Table 1, the recovery rates in portable reader mode ranged from 90.3% to 100.3%, with a coefficient of variation (CV) between 1.53% and 19.79%. In photothermal instrument mode, recovery rates varied from 95.0% to 102.7%, and CV values ranged from 1.82% to 15.47%. Validation by ELISA confirmed recovery rates of 94.3% to 101.3%, with CVs ranging from 1.11% to 11.93%. Statistical analysis demonstrated that this method offers high stability and minimal data dispersion. These findings indicate that the dual-mode quantitative immunochromatographic assay developed in this study is suitable for reliable CIP residue detection in real samples.

## 4. Discussion

In summary, this study developed a PVP@Pd NPs-based immunochromatographic assay to achieve dual-signal output through a portable reader and a photothermal instrument. The entire process, from loading the sample to obtaining the final signal output, can be completed within 15 min. With optimized experimental parameters, the method could detect the concentration of CIP in the portable reader mode (Mode 1) in the range of 0.1 to 100.0 ng/mL with an LOD of 0.1 ng/mL. In the photothermal instrument mode (Mode 2), the detection range was extended to 500.0 ng/mL and the LOD was maintained. The sensitivity and accuracy of the immunochromatographic method were evaluated by measuring the concentrations of CIP in real samples. The experimental results show that the proposed method has excellent specificity, good stability and wide applicability, making it suitable for CIP detection in different types of fish products. This study provides a novel strategy to achieve the high-sensitivity and rapid detection of CIP in fish products which has great application potential, and also lays a valuable foundation for detection in other real samples in the future.

## Figures and Tables

**Table 1:** Comparison of detection results between modes and ELISA in real samples.

Sample	Add Concentration/(ng/mL)	Mode 1 Test Results			Mode 2 Test Results			ELISA Test Results		
		Mean (ng/mL)	Recovery (%)	CV (%)	Mean (ng/mL)	Recovery (%)	CV (%)	Mean (ng/mL)	Recovery (%)	CV (%)
*Larimichthys crocea*	0.00	ND ^1^	-	-	ND	-	-	ND	-	-
	10.00	10.03	100.30	14.97	10.27	102.70	11.50	9.50	95.00	9.48
	20.00	19.60	98.00	6.83	20.03	100.30	5.97	19.67	98.30	3.15
*Carassius auratus*	0.00	ND	-	-	ND	-	-	ND	-	-
	10.00	10.00	100.00	7.69	9.50	95.00	15.47	10.13	101.30	7.74
	20.00	19.80	99.00	1.53	19.60	98.00	4.00	19.87	99.40	7.39
*Micropterus salmoides*	0.00	ND	-	-	ND	-	-	ND	-	-
	10.00	9.90	99.00	18.93	9.90	99.00	13.9	9.57	95.70	10.09
	20.00	19.30	96.50	3.47	19.47	97.40	4.50	19.30	96.50	3.32
*Channa argus*	0.00	ND	-	--	ND	-	-	ND	-	-
	10.00	9.630	96.30	14.44	9.47	94.70	10.25	10.13	101.30	6.71
	20.00	19.90	99.50	6.75	19.47	97.40	1.82	19.77	98.80	2.69
*Parabramis pekinensis*	0.00	ND	-	-	ND	-	-	ND	-	-
	10.00	9.030	90.30	19.79	10.13	101.30	9.74	9.43	94.30	11.93
	20.00	19.30	96.50	5.27	19.83	99.20	2.57	19.63	98.20	1.11

^1^ ND: Not Detected.

## Data Availability

The original contributions presented in the study are included in the article/Supplementary Material, further inquiries can be directed to the corresponding author.

## Supplementary Materials

The following supporting information can be downloaded at: https://www.mdpi.com/article/10.3390/foods14071132/s1, Figure S1: S-shaped curve of Enzyme-Linked Immunosorbent Assay of ciprofloxacin in PBS; Table S1: Optimal working conditions of ELISA in PBS (pH 7.4); Table S2: Comparison of ciprofloxacin detection methods [23,35,45,46,47,48].
