# Supplementary material for: Dual-Mode Quantitative Immunochromatographic Assay for Highly Sensitive On-Site Detection of Ciprofloxacin in Fish Products
Source: Foods. 2025 Mar 25;14(7):1132. doi: 10.3390/foods14071132 (PMC11989092; doi:10.3390/foods14071132)
Supplement: Supplementary file 1 [file foods-14-01132-s001.zip › foods-3545651-supplementary.pdf]

Dual-Mode Quantitative Immunochromatographic Assay for Highly Sensitive On-Site Detection of Ciprofloxacin in Fish Products

Junqi Shen [1], Zhengyi Cai [1], Cheng Zhang [1], Xinyue Feng [1], Chenzhi Zhang [1], Huan Zhao [1], Chuanlin Yin [1,2,*], Bo Wang [3], Xiaoping Yu [1] and Biao Zhang [1,*]

1 Key Laboratory of Microbiological Metrology, Measurement & Bio-Product Quality Security, State Administration for Market Regulation, College of Life Sciences, China Jiliang University, Hangzhou 310018, China; shenjunqi@cjlu.edu.cn (J.S.); s24090710003@cjlu.edu.cn (Z.C.); 19548195033@163.com (C.Z.); fxy18767053369@163.com (X.F.); luocheng2026@163.com (C.Z.); kroyihuan@163.com (H.Z.); yxp@cjlu.edu.cn (X.Y.)

2 College of Life Sciences, China Jiliang University, Hangzhou 310018, China

3 College of Food Science and Engineering, Yangzhou University, Yangzhou 225009, China; wb@yzu.edu.cn

* Correspondence: chuanlinyin@cjlu.edu.cn (C.Y.); zhangbiao9129@163.com (B.Z.)

Contents

Table S1   **Table S2**

Figure S1

Table S1. Optimal working conditions of ELISA in PBS (pH 7.4).

Factor	Conditions
Amount of antibody working fluid	50 μL/well
Amount of standard working fluid	50 μL/well
Blocking solution	0.5% skim milk powder
Competition time	30 min
Time required	About 3 hours

Table S2. Comparison of Ciprofloxacin detection methods.

Methods	Material or system	Linear range	LOD	Reference
HPLC	-	0.1–100 ng/mL.	0.075 ng/mL	[1]
Fluorescence	Eu MOFs	0- 39,760.8 ng/mL	16.70 ng/mL	[2]
Immunosensor	Quantum dot microsphere	0.1 -100 ng/mL	0.05 ng/mL	[3]
Electrochemical sensor	Co-Fe-PBA@CN	1.657–99,402 ng/mL	0.2448 ng/mL	[4]
Optical sensing	responsive photonic crystal	0.0331–33,134 ng/mL	0.0166 ng/mL	[5]
ELISA	-	0.12-68.40 ng/ml	0.06 ng/ml	[6]
Dual-Mode Photothermal Quantitative Immunochromatographic Assay	PVP@Pd NPs	1-500 ng/mL	0.1 ng/mL	This work

Reference:

1. Gezahegn, T.; Tegegne, B.; Zewge, F.; Chandravanshi, B.S. Salting-out assisted liquid–liquid extraction for the determination of ciprofloxacin residues in water samples by high performance liquid chromatography–diode array detector. BMC Chemistry 2019, 13, 28, doi:10.1186/s13065-019-0543-5.
2. Meng, S.; Liu, J.X.; Yang, Y.Y.; Mao, S.; Li, Z. Lanthanide MOFs based portable fluorescence sensing platform: Quantitative and visual detection of ciprofloxacin and Al3+. Science of the Total Environment 2024, 922, doi:10.1016/j.scitotenv.2024.171115.
3. Liu, J.; Wang, B.; Huang, H.C.; Jian, D.; Lu, Y.A.; Shan, Y.K.; Wang, S.Y.; Liu, F. Quantitative ciprofloxacin on-site rapid detections using quantum dot microsphere based immunochromatographic test strips. Food Chemistry 2021, 335, doi:10.1016/j.foodchem.2020.127596.
4. Umesh, N.M.; Jesila, J.A.A.; Wang, S.F. Amperometric detection of antibiotic drug ciprofloxacin using cobalt-iron Prussian blue analogs capped on carbon nitride. Microchimica Acta 2022, 189, doi:10.1007/s00604-021-05061-z.
5. Zhang, R.; Wang, Y.; Yu, L.P. Specific and ultrasensitive ciprofloxacin detection by responsive photonic crystal sensor. Journal of Hazardous Materials 2014, 280, 46-54, doi:10.1016/j.jhazmat.2014.07.032.

6. Fan, G.Y.; Yang, R.S.; Jiang, J.Q.; Chang, X.Y.; Chen, J.J.; Qi, Y.H.; Wu, S.X.; Yang, X.F. Development of a class-specific polyclonal antibody-based indirect competitive ELISA for detecting fluoroquinolone residues in milk. Journal of Zhejiang University-Science B 2012, 13, 545-554, doi:10.1631/jzus.B1200001.

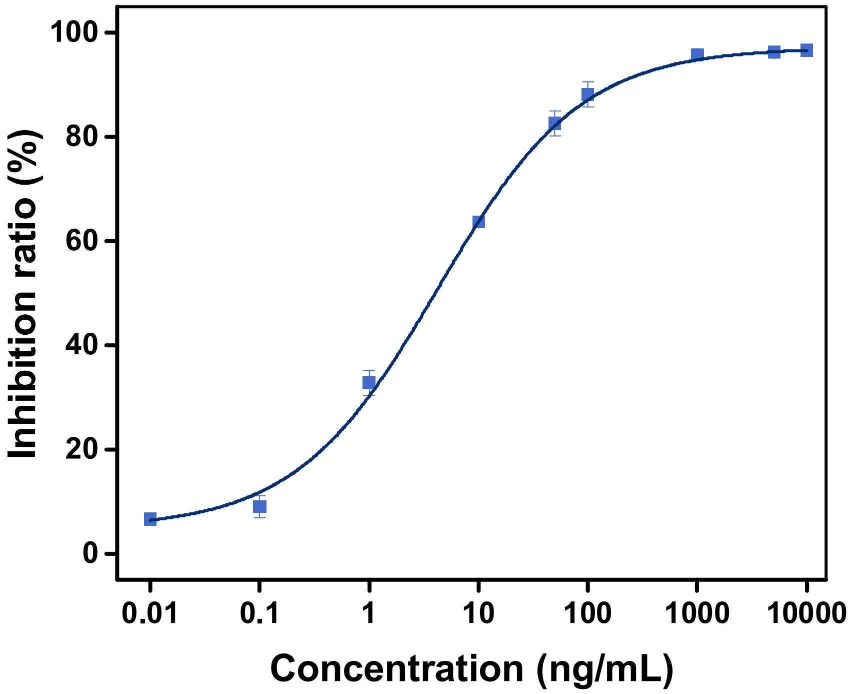

Figure S1. S-shaped curve of Enzyme-linked immunosorbent assay of ciprofloxacin in PBS.
